# Comparative effectiveness of teriflunomide and ocrelizumab on smoldering activity in multiple sclerosis: an observational study in the Swiss Multiple Sclerosis Cohort

**DOI:** 10.1007/s00415-025-13221-x

**Published:** 2025-07-02

**Authors:** Alessandro Cagol, Sabine Schaedelin, Mario Ocampo-Pineda, Pascal Benkert, Lester Melie-Garcia, Ludovico Luchetti, Özgür Yaldizli, Johanna Oechtering, Marcus D’Souza, Bettina Fischer-Barnicol, Stefanie Müller, Sebastian Finkener, Jochen Vehoff, Giulio Disanto, Andrew Chan, Caroline Pot, Chiara Zecca, Tobias Derfuss, Johanna M. Lieb, Michael Diepers, Franca Wagner, Renaud Du Pasquier, Patrice H. Lalive, Emanuele Pravatà, Olaf Chan-Hi Kim, Robert Hoepner, Patrick Roth, Claudio Gobbi, David Leppert, Marco Battaglini, Ludwig Kappos, Maria Pia Sormani, Jens Kuhle, Cristina Granziera, for the Swiss MS Cohort

**Affiliations:** 1 Translational Imaging in Neurology (ThINk) Basel, Department of Biomedical Engineering, Faculty of Medicine, University Hospital Basel and University of Basel, Hegenheimermattweg 167b, 4123, Allschwil, Basel, Switzerland; 2 Multiple Sclerosis Centre, Departments of Neurology, Clinical Research and Biomedicine, University Hospital Basel and University of Basel, Basel, Switzerland; 3 Research Center for Clinical Neuroimmunology and Neuroscience Basel (RC2NB), University Hospital Basel and University of Basel, Basel, Switzerland; 4 Dipartimento di Scienze della Salute, Università degli Studi di Genova, Genoa, Italy; 5 Department of Clinical Research, University Hospital Basel, University of Basel, Basel, Switzerland; 6 Clinic of Neurology, HOCH Health Ostschweiz, Cantonal Hospital St. Gallen, Academic and Research Hospital, St. Gallen, Switzerland; 7 Department of Neurology, Cantonal Hospital Aarau, Aarau, Switzerland; 8 Neurology Department, Neurocenter of Southern Switzerland, EOC, Lugano, Switzerland; 9 Department of Neurology, Inselspital, Bern University Hospital and University of Bern, Bern, Switzerland; 10 Division of Neurology, Department of Clinical Neurosciences, Lausanne University Hospital (CHUV) and University of Lausanne, Lausanne, Switzerland; 11 Department of Clinical Neurosciences, Division of Neurology, Geneva University Hospitals and Faculty of Medicine, Geneva, Switzerland; 12 Faculty of Biomedical Sciences, Università della Svizzera Italiana, Lugano, Switzerland; 13 Division of Diagnostic and Interventional Neuroradiology, Clinic for Radiology and Nuclear Medicine, University Hospital Basel, University of Basel, Basel, Switzerland; 14 Department of Diagnostic and Interventional Neuroradiology, Cantonal Hospital Aarau, Aarau, Switzerland; 15 Department of Diagnostic and Interventional Neuroradiology, Inselspital, Bern University Hospital and University of Bern, Bern, Switzerland; 16 Division of Radiology, Lausanne University Hospital (CHUV) and University of Lausanne, Lausanne, Switzerland; 17 Department of Neuroscience, Imaging and Clinical Sciences, Institute of Advanced Biomedical Technologies, University of Chieti, 66100 Chieti, Italy; 18 Department of Radiology, Cantonal Hospital St. Gallen, St. Gallen, Switzerland; 19 Department of Neurology, University of Zurich, University Hospital of Zurich, Zurich, Switzerland; 20 IRCCS Ospedale Policlinico San Martino, Genoa, Italy; 21 Department of Medicine, Surgery and Neurosciences, University of Siena, Siena, Italy; 22 SIENA Imaging SRL, Siena, Italy

**Keywords:** Disease-modifying therapies, Ocrelizumab, Teriflunomide, PIRA, Smoldering MS, PRLs

## Abstract

**Background:**

This study aimed to compare the effects of teriflunomide and ocrelizumab on clinical and MRI endpoints related to smoldering activity in relapsing–remitting multiple sclerosis (RRMS).

**Methods:**

In this observational, longitudinal, multicenter study, we included 128 people with RRMS (pwRRMS) treated with teriflunomide and 495 treated with ocrelizumab. Outcomes included time to progression independent of relapse activity (PIRA). In a subset, we also assessed brain volume loss (BVL), longitudinal changes in diffusion tensor imaging (DTI) metrics, and the burden of paramagnetic rim lesions (PRLs). Propensity score matching was used for between-group comparisons.

**Results:**

Over a median follow-up of 3.1 years in the ocrelizumab group and 1.9 years in the teriflunomide group, there were no significant differences in the risk of PIRA (HR for teriflunomide vs. ocrelizumab: 0.80 [95%-CI:0.40–1.60]; p = 0.53). PwRRMS treated with teriflunomide exhibited lower annualized rates of BVL (−0.80 [95%-CI: −0.91; −0.69] vs. −1.06 [95%-CI: −1.25; −0.86]; p = 0.025) and gray matter volume loss (−0.92 [95%-CI: −1.05; −0.79] vs. −1.20 [95%-CI: −1.43; −0.97]; p = 0.035). No differences were observed in DTI metrics or PRL count.

**Conclusions:**

This real-world study suggests that teriflunomide shows similar efficacy to ocrelizumab on smoldering activity, with a potentially greater effect in reducing BVL. Further research is needed to confirm these findings and understand their long-term implications.

**Supplementary Information:**

The online version contains supplementary material available at 10.1007/s00415-025-13221-x.

## Introduction

Multiple sclerosis (MS) is an immune-mediated neurological disease characterized by inflammation, demyelination, and neurodegeneration, ultimately leading to progressive disability. Traditionally, relapsing–remitting MS (RRMS) was thought to be defined solely by acute inflammatory events interspersed with periods of stability. However, it is now increasingly recognized that smoldering disease processes play a critical role in RRMS [1, 2]. A key concept in this evolving understanding is progression independent of relapse activity (PIRA), which refers to the gradual accumulation of disability driven by underlying disease processes that are unrelated to acute inflammatory events [3]. PIRA has been shown to occur frequently in people with RRMS (pwRRMS), where it is the primary determinant of disability accrual in the modern treatment era [3, 4].

The smoldering mechanisms underlying disease progression include non-resolving inflammation, neurodegeneration, oxidative stress, and mitochondrial dysfunction, which may occur independently or interact with each other [1]. In addition, the failure of compensatory mechanisms, such as remyelination and neuroplasticity, together with aging exacerbate these processes [1]. Some of these smoldering processes can be detected in vivo using advanced MRI biomarkers derived from both conventional and non-conventional techniques. These include the estimation of global and regional brain volume loss (BVL), particularly in gray matter structures, which serves as a proxy for the overall neurodegenerative burden of the disease [5]. Paramagnetic rim lesions (PRLs) are another important biomarker, gaining increasing importance for their ability to identify chronic active lesions, which are associated with worse clinical outcomes [6]. Diffusion MRI, which is sensitive to microstructural tissue integrity, provides indirect measures of demyelination and axonal loss [7, 8]. Notably, all these MRI measures have demonstrated sensitivity to the pathological changes occurring in patients with PIRA [9–13].

Together, the smoldering neurodegenerative and chronic inflammatory processes shape the clinical trajectories of MS, emphasizing the need for therapeutic strategies that address the complex nature of the disease. The currently approved disease-modifying therapies (DMTs) vary in their efficacy in providing neuroprotection. Teriflunomide, an oral immunomodulator approved for the treatment of relapsing forms of MS, targets dihydroorotate dehydrogenase, a mitochondrial enzyme involved in pyrimidine synthesis, thereby reducing the activity of proliferating B and T cells. In phase II and phase III clinical trials, teriflunomide showed improvements over placebo in annualized relapse rate (ARR), MRI lesions, and disability accumulation [14]. Additionally, teriflunomide has been shown to reduce BVL in both clinical trials and real-world studies, suggesting a potential effect on neurodegenerative processes [14]. Ocrelizumab, a recombinant humanized anti-CD20 monoclonal antibody, is approved for the treatment of both relapsing forms of MS and primary progressive MS (PPMS). In RRMS, ocrelizumab has demonstrated efficacy in reducing ARR, MRI lesions, and disability accumulation as compared to interferon beta-1a [15]. However, while ocrelizumab demonstrated superiority over placebo in reducing BVL in patients with PPMS, it did not show greater efficacy compared to interferon beta-1a in pwRRMS [15]. Notably, a post-hoc analysis of the pooled OPERA I and II trials showed that ocrelizumab is effective in reducing the incidence of PIRA compared to interferon beta-1a [16].

To date, no head-to-head comparisons have been conducted between teriflunomide and ocrelizumab, and the impact of these treatments on clinical and MRI measures of smoldering activity remains poorly explored.

In this study, we aimed to compare the association of teriflunomide and ocrelizumab with PIRA and MRI measures of smoldering disease activity in an observational multicenter cohort of pwRRMS.

## Patients and methods

### Participants and study design

In this observational study, participants were selected from the Swiss Multiple Sclerosis Cohort (SMSC), a prospective, multicenter study conducted across eight Swiss academic centers that features standardized collection of demographic, clinical, and MRI data [17]. For our investigation, we retrospectively included all adult SMSC participants diagnosed with RRMS who were treated with either teriflunomide or ocrelizumab during follow-up. We analyzed all longitudinal clinical and MRI examinations for each patient from the initiation of teriflunomide or ocrelizumab treatment, censoring data at the last visit, or at treatment discontinuation. Patients who received both treatments during SMSC follow-up were included only with data corresponding to the first treatment chronologically.

Specific analysis sets, with propensity score matching between treatment groups, were defined for each study endpoint based on data availability. The study endpoints included:

1. Time to PIRA, analyzed in the entire study cohort.
2. Global and regional BVL, analyzed in participants with at least one brain MRI scan including three-dimensional (3D) magnetization-prepared rapid gradient echo (MPRAGE).
3. Longitudinal changes in diffusion tensor imaging (DTI) metrics within white matter lesions (WMLs), normal-appearing white matter (NAWM), and cerebral cortex, analyzed in participants with at least one brain MRI scan including diffusion-weighted imaging.
4. Longitudinal changes in paramagnetic rim lesion (PRL) burden, analyzed in participants with at least one brain MRI scan including 3D segmented echo planar imaging (EPI).

**Fig. 1:**
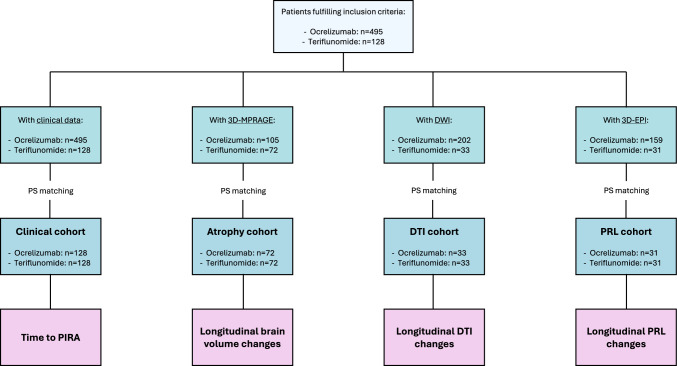
Study design. Abbreviations: 3D-EPI = three-dimensional echo planar imaging; 3D-MPRAGE = three-dimensional magnetization-prepared rapid gradient echo; DTI = diffusion tensor imaging; DWI = diffusion-weighted imaging; PIRA = progression independent of relapse activity; PRL = paramagnetic rim lesion; PS = propensity score

The study design is graphically displayed in Fig. 1. The study was approved by the local ethics committee, and all patients provided written informed consent before study entry.

### Clinical data

All participants underwent regular clinical evaluations, conducted at least annually. Standardized assessments included the calculation of the Expanded Disability Status Scale (EDSS) score (https://www.neurostatus.net/), performed by certified raters.

The occurrence of PIRA during follow-up was defined as an increase in EDSS score (≥ 1.5, ≥ 1.0, or ≥ 0.5 points if baseline EDSS was 0, 1.0–5.5, or > 5.5, respectively) using a roving baseline [18], confirmed at least after 6 months, in the absence of relapses (1) between the EDSS increase and the preceding reference visit (conducted ≥ 90 days before the EDSS increase) and (2) between the EDSS increase and its confirmation [3].

### MRI acquisition and analysis

Brain MRI scans were performed at each center with protocols optimized to ensure a homogeneous signal-to-noise ratio. Protocol details are provided in eTables 1–3.

Segmentation of T2-hyperintense WMLs on 3D fluid attenuated inversion recovery (FLAIR) images was performed using a deep learning-based tool [19], followed by manual correction.

Brain volumetric measurements were obtained on 3D-MPRAGE images using the longitudinal pipeline of *SAMSEG*, with 3D-FLAIR images included as an additional input to optimize automatic segmentation [20]. The volumes of interest considered in the study included total brain volume, cortical volume, thalamic volume, and total gray matter volume. Total intracranial volume (TIV) was also calculated to account for between-subject differences in head size in the statistical analyses.

DTI metrics were derived from diffusion-weighted images after denoising and correction for ringing artifacts, eddy current distortions, misalignments, and bias field [21]. Fractional anisotropy (FA), radial diffusivity (RD), mean diffusivity (MD), and axial diffusivity (AD) maps were generated using *MRtrix* [21]. For each map, mean values within WMLs, NAWM, and cerebral cortex were derived.

The presence of PRLs was assessed by an experienced rater (A.Ca.), who was blinded to patient identity. PRLs were defined as discrete FLAIR-hyperintense lesions either completely or partially encircled by a rim of paramagnetic signal, clearly evident in at least one contrast between unwrapped phase and quantitative susceptibility mapping (QSM), as previously described [10]. High inter-rater agreement in PRL detection was previously demonstrated in the same cohort [10].

In patients with PIRA, we assessed the accumulation of new or enlarged T2-hyperintense WMLs to identify events occurring without accompanying MRI activity, thereby fulfilling the criteria for progression independent of relapse and MRI activity (PIRMA) [3, 22]. This analysis was conducted using a semi-automated approach to systematically compare all 3D-FLAIR images acquired during routine follow-up in the SMSC [23].

### Statistical analysis

All statistical analyses were conducted using *R* (*version 4.3.1*).

To mitigate bias from the non-random assignment to treatments in the SMSC study, we performed 1:1 propensity score matching between treatment groups. The matching criteria included the following variables at baseline: age, sex, disease duration, number of previous DMTs, number and volume of WMLs, raw and Z score levels of serum neurofilament light chain (sNfL) [24], EDSS score, and time under current treatment. Separate matched sets were constructed for each endpoint to maximize sample size.

We compared the incidence of PIRA between groups using Cox proportional hazard models to assess time to PIRA. Longitudinal changes in brain volumes, DTI metrics, and PRL burden were analyzed using mixed-effect models. These models included an interaction term between treatment group and time to examine group differences in longitudinal changes, with participants included as random intercepts. When modeling BVL, scanner magnetic field strength and TIV were included as additional covariates. Time was log-transformed to derive the annualized percentage change (APC) in MRI metrics. Model assumptions were assessed through visual inspection of residuals.

For time to PIRA, the following sensitivity analyses were performed: (1) censoring patients at the same time as their matching pair rather than at their last observation (pairwise censoring); (2) including only PIRA episodes where the initial EDSS increase occurred after treatment initiation; and (3) exploring time to PIRMA.

For BVL, the following sensitivity analyses were conducted: (1) including baseline age, sex, and disease duration, along with their interaction with time, as covariates in the mixed-effect models; (2) including ARR during MRI follow-up, along with its interaction with time, as covariates in the mixed-effect models; (3) including only patients with at least two MRI scans for volumetric analysis; and (4) including only scans obtained at least 6 months after treatment initiation.

All statistical analyses were conducted by a statistician (S.S.) who was not involved in the MRI analyses, while the authors conducting the MRI analyses did not have access to patient information, including treatment details.

## Results

A total of 623 pwRRMS met the inclusion criteria and were enrolled in the study. The key demographic and clinical characteristics are summarized in Table 1. Separate match sets were created for each outcome of interest, and the baseline MRI characteristics showed no significant differences between the propensity score-matched groups (Table 2).

**Table 1:** Key baseline characteristics of the cohort

n	623
Age, mean (SD), years	43.1 (12.5)
Females, No. (%)	408 (65.5)
Disease duration, median [IQR], years	9.2 [3.6; 16.7]
EDSS, median [IQR]	2.5 [1.5; 3.5]
Number of previous DMTs, median [IQR]	1 [0; 3]
Time under current treatment, median [IQR], years	2.2 [0.0; 5.1]
sNfL, median [IQR], pg/ml	10.2 [7.3; 14.8]
sNfL Z-score, median [IQR]	0.8 [−0.1; 1.7]
T2LV, median [IQR], ml	7.4 [3.4; 14.2]
T2L count, median [IQR]	32.0 [21.7; 42.0]
SMSC centers	
– Aarau, No. (%) – Basel, No. (%) – Bern, No. (%) – Geneva, No. (%) – Lausanne, No. (%) – Lugano, No. (%) – St. Gallen, No. (%) – Zurich, No. (%)	71 (11.4) 315 (50.6) 17 (2.7) 64 (10.3) 46 (7.4) 52 (8.3) 42 (6.7) 16 (2.6)

*DMT* disease modifying treatment, *EDSS*  Expanded Disability Status Scale,* IQR* interquartile range,* SD* standard deviation, *SMSC* Swiss Multiple Sclerosis Cohort, *sNfL* serum neurofilament light chain,* T2L* T2-lesion, *T2LV* T2-lesion volume

**Table 2:** MRI characteristics at baseline

	Ocrelizumab	Teriflunomide	Comparison
Atrophy cohort			
n	72	72	
Brain parenchymal fraction, median [IQR]	0.69 [0.66;0.71]	0.68 [0.65;0.73]	^a^p = 0.85; SMD = 0.067
Cortical fraction, median [IQR]	0.31 [0.29;0.33]	0.31 [0.29;0.33]	^a^p = 0.99; SMD = 0.028
Thalamic fraction, median [IQR]	0.01 [0.01;0.01]	0.01 [0.01;0.01]	^a^p = 0.53; SMD = 0.217
Gray matter fraction, median [IQR]	0.40 [0.38;0.43]	0.41 [0.38;0.44]	^a^p = 0.56; SMD = 0.088
DTI cohort			
n	33	33	
AD WMLs, median [IQR], mm^2^/s	0.00149 [0.00141;0.00158]	0.00145 [0.00141;0.00157]	^a^p = 0.77; SMD = 0.188
AD NAWM, median [IQR], mm^2^/s	0.00106 [0.00105;0.00108]	0.00107 [0.00105;0.00109]	^a^p = 0.79; SMD = 0.023
AD Cortex, median [IQR], mm^2^/s	0.00106 [0.00104;0.00109]	0.00106 [0.00103 0.00108]	^a^p = 0.69; SMD = 0.057
FA WMLs, median [IQR]	0.300 [0.277;0.320]	0.303 [0.287;0.340]	^a^p = 0.24; SMD = 0.364
FA NAWM, median [IQR]	0.376 [0.363;0.388]	0.386 [0.375;0.396]	^a^p = 0.12; SMD = 0.440
FA Cortex, median [IQR]	0.170 [0.158;0.175]	0.169 [0.163;0.173]	^a^p = 0.88; SMD = 0.237
MD WMLs, median [IQR], mm^2^/s	0.00113 [0.00107;0.00121]	0.00109 [0.00103;0.00121]	^a^p = 0.31; SMD = 0.274
MD NAWM, median [IQR], mm^2^/s	0.00074 [0.00073;0.00076]	0.00074 [0.00073;0.00076]	^a^p = 0.88; SMD = 0.201
MD Cortex, median [IQR], mm^2^/s	0.00091 [0.00090;0.00093]	0.00091 [0.00088;0.00092]	^a^p = 0.39; SMD = 0.173
RD WMLs, median [IQR], mm^2^/s	0.00095 [0.00091;0.00102]	0.00090 [0.00084;0.00102]	^a^p = 0.21; SMD = 0.307
RD NAWM, median [IQR], mm^2^/s	0.00058 [0.00057;0.00060]	0.00057 [0.00056;0.00060]	^a^p = 0.57; SMD = 0.271
RD Cortex, median [IQR], mm^2^/s	0.00083 [0.00082;0.00085]	0.00083 [0.00081;0.00085]	^a^p = 0.30; SMD = 0.214
PRL cohort			
n	31	31	
PRL count, median [IQR]	0 [0; 2]	1 [0; 3.25]	^a^p = 0.17; SMD = 0.036

Brain parenchymal fraction, cortical fraction, thalamic fraction, and gray matter fraction were obtained by dividing total brain volume, cortical volume, thalamic volume, and gray matter volume by the total intracranial volume

^a^Obtained with Mann–Whitney U test

*AD* axial diffusivity, *DTI* diffusion tensor imaging, *FA* fractional anisotropy, *IQR* interquartile range, *MD* mean diffusivity, *NAWM* normal-appearing white matter, *PRL* paramagnetic rim lesions, *RD* radial diffusivity, *SMD* standardized mean difference, *SD* standard deviation, *WMLs* white matter lesions

### Incidence of PIRA

**Fig. 2:**
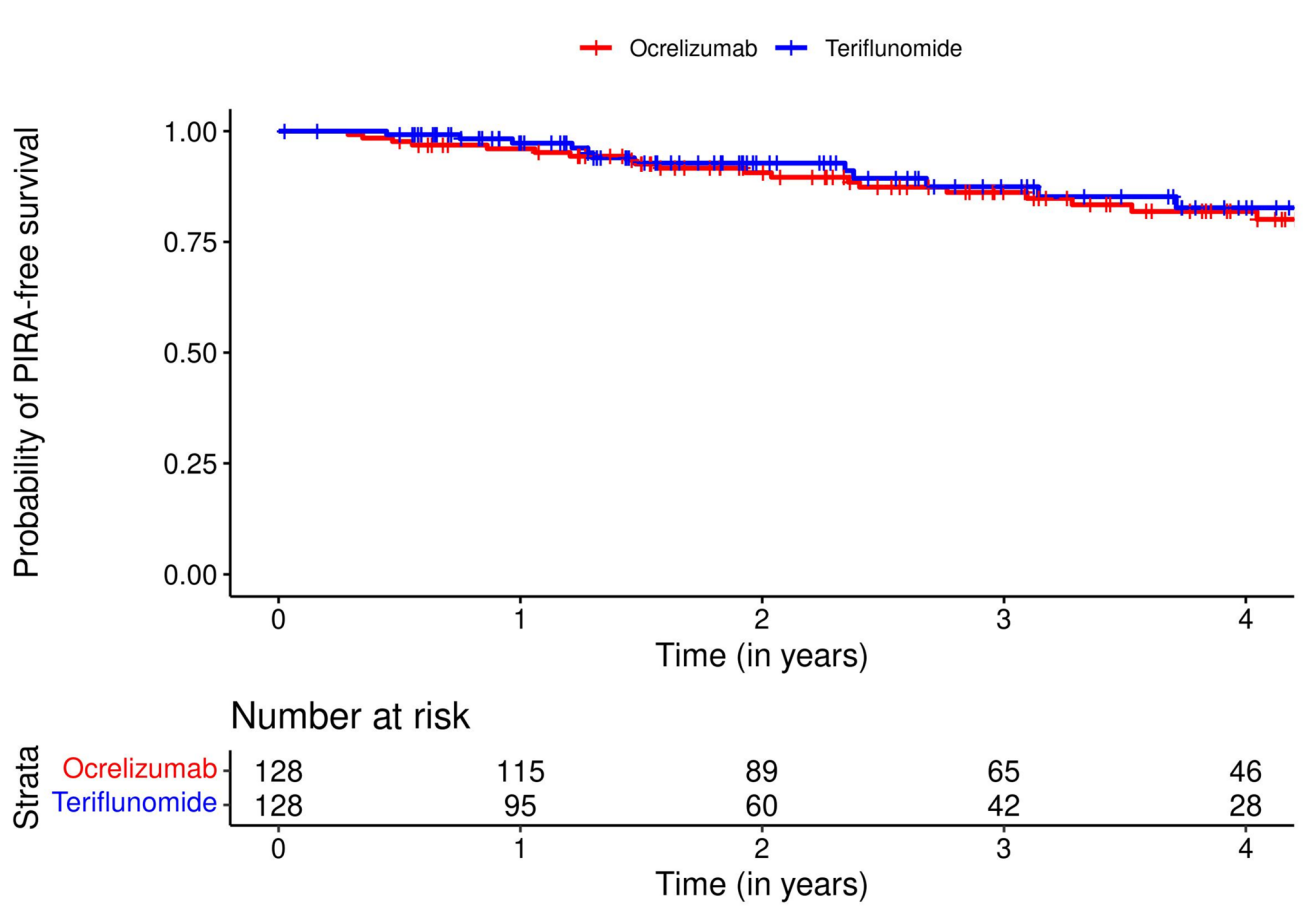
Probability of PIRA-free survival. Abbreviations: PIRA = progression independent of relapse activity

Baseline characteristics of the clinical cohort before and after propensity score matching are reported in eTable 4. A total of 128 pwRRMS per treatment group contributed to the analysis. The median (IQR) follow-up time was 3.1 (1.4; 4.6) years for the ocrelizumab group and 1.9 (1.0; 3.7) years for the teriflunomide group. During the observation period, there were 35 PIRA events, with 21 occurring in the ocrelizumab group and 14 in the teriflunomide group. The time to PIRA was not significantly different between treatment groups (hazard ratio [HR] for teriflunomide vs. ocrelizumab: 0.80 [95% CI: 0.40; 1.60]; p = 0.53). Kaplan–Meier curves for time to PIRA are displayed in Fig. 2.

Results remained consistent in sensitivity analyses applying pairwise censoring (HR for teriflunomide vs. ocrelizumab: 0.90 [95% CI: 0.37; 2.21]; p = 0.82) and excluding PIRA events with onset before treatment initiation (HR for teriflunomide vs. ocrelizumab: 0.93 [95% CI: 0.43; 2.02]; p = 0.86). 32 out of 35 PIRA events qualified as PIRMA; the time to PIRMA was not different between treatment groups (HR for teriflunomide vs. ocrelizumab: 0.75 [95% CI: 0.36; 1.56]; p = 0.44).

### Brain volume loss

**Fig. 3:**
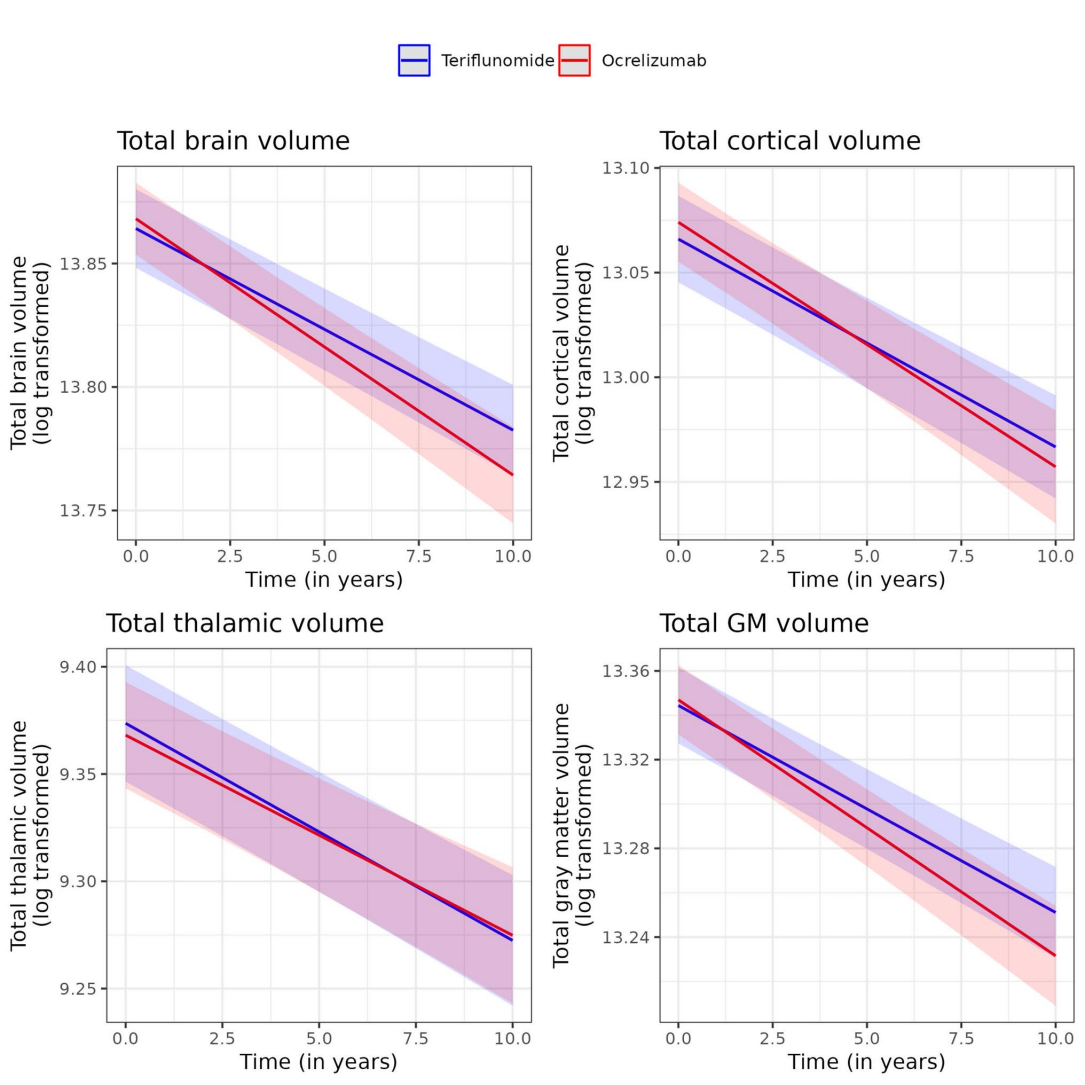
Between group comparison of brain volume loss rates

Baseline characteristics of the brain atrophy cohort before and after propensity score matching are reported in eTable 5. A total of 72 pwRRMS per treatment group contributed to the analysis. The median (IQR) follow-up time was 1.7 (0.6; 2.5) years for the ocrelizumab group and 1.0 (0.0; 3.8) years for the teriflunomide group. Compared to pwRRMS treated with ocrelizumab, those treated with teriflunomide exhibited reduced rates of total BVL (APC: −0.80 [95% CI: −0.91; −0.69] vs. −1.06 [95% CI: −1.25; −0.86]; p = 0.025) and gray matter volume loss (APC: −0.92 [95% CI: −1.05; −0.79] vs. −1.20 [95% CI: −1.43; −0.97]; p = 0.035) during the observation period (Fig. 3). There were no significant differences in the rates of cortical volume and thalamic volume loss between groups (Table 3).

**Table 3:** Longitudinal changes in MRI metrics

	Ocrelizumab (APC)	Teriflunomide (APC)	Comparison
Atrophy cohort			
n	72	72	
Total brain volume change, mean (95% CI)	−1.06 (−1.25; −0.86)	−0.80 (−0.91; −0.69)	^a^**p = 0.025**
Cortical volume change, mean (95% CI)	−1.24 (−1.51; −0.97)	−0.97 (−1.12, −0.82)	^a^p = 0.09
Thalamic volume change, mean (95% CI)	−0.97 (−1.23; −0.71)	−1.04 (−1.19; −0.90)	^a^p = 0.64
Gray matter volume change, mean (95% CI)	−1.20 (−1.43; −0.97)	−0.92 (−1.05; −0.79)	^a^**p = 0.035**
No. of MRI follow-ups, median [IQR]	2.5 [2.0;3.0]	2.0 [1.0;4.3]	^b^p = 0.81; SMD = 0.295
Follow-up time, median [IQR], years	1.7 [0.6; 2.5]	1.0 (0.0; 3.8)	^b^p = 0.83; SMD = 0.246
DTI cohort			
n	33	33	
AD WMLs change, mean (95% CI)	−0.05 (−0.72; 0.62)	0.79 (0.15; 1.44)	^a^p = 0.08
AD NAWM change, mean (95% CI)	0.10 (−0.18; 0.38)	−0.00 (−0.27; 0.26)	^a^p = 0.60
AD Cortex change, mean (95% CI)	−0.30 (−0.68; 0.09)	0.08 (−0.28; 0.44)	^a^p = 0.17
FA WMLs change, mean (95% CI)	−0.04 (−1.29; 1.20)	0.23 (−0.94; 1.41)	^a^p = 0.76
FA NAWM change, mean (95% CI)	0.62 (−0.11; 1.34)	−0.27 (−0.95; 0.40)	^a^p = 0.08
FA Cortex change, mean (95% CI)	−0.43 (−1.36; 0.52)	0.33 (−0.58; 1.24)	^a^p = 0.26
MD WMLs change, mean (95% CI)	0.14 (−0.65; 0.93)	0.84 (0.08; 1.60)	^a^p = 0.21
MD NAWM change, mean (95% CI)	−0.11 (−0.55; 0.33)	0.10 (−0.31; 0.51)	^a^p = 0.50
MD Cortex change, mean (95% CI)	−0.24 (−0.56; 0.09)	0.01 (−0.30; 0.32)	^a^p = 0.29
RD WMLs change, mean (95% CI)	0.30 (−0.69; 1.30)	0.89 (−0.07; 1.85)	^a^p = 0.41
RD NAWM change, mean (95% CI)	−0.29 (−0.92; 0.34)	0.19 (−0.40; 0.79)	^a^p = 0.28
RD Cortex change, mean (95% CI)	−0.20 (−0.52; 0.12)	−0.06 (−0.36; 0.25)	^a^p = 0.52
No. of MRI follow-ups, median [IQR]	3 [2; 4]	3 [2; 4]	^b^p = 0.75; SMD = 0.050
Follow-up time, median [IQR], years	2.0 [1.7; 2.9]	2.0 [1.0; 2.9]	^b^p = 0.91; SMD = 0.104
PRL cohort			
n	31	31	
Longitudinal changes over follow-up:			
– Subjects with new PRLs, No (%)	1 (3)	2 (6)	^a^p = 0.96
– Subjects with resolving PRLs, No (%)	0 (0)	0 (0)	
No. of MRI follow-ups, median [IQR]	2 [2; 3]	3 [2; 4]	^b^p = 0.25; SMD = 0.171
Follow-up time, median [IQR], years	1.5 [1.0; 2.2]	2.0 [1.2; 2.9]	^b^p = 0.07; SMD = 0.452

^a^Obtained from the mixed-effect models

^b^Obtained with Mann–Whitney U test

*AD* axial diffusivity, *APC* annualized percentage change, *DTI* diffusion tensor imaging, *FA* fractional anisotropy, *IQR* interquartile range, *MD* mean diffusivity, *MRI* magnetic resonance imaging, *NAWM* normal-appearing white matter, *PRL* paramagnetic rim lesions, *RD* radial diffusivity, *SMD* standardized mean difference, *SD* standard deviation, *WMLs* white matter lesions

Significant *p*-values are displayed in bold

A significantly lower rate of BVL in patients treated with teriflunomide was confirmed in sensitivity analyses adjusting for baseline age, sex, and disease duration, as well as for ARR during the observation period, and in the subset of patients with at least 2 MRI time points (eTables 6–8). When including only scans performed at least 6 months after treatment initiation, the between-group differences in rates of BVL lost statistical significance (eTable 9).

### DTI metrics

Baseline characteristics of the DTI cohort before and after propensity score matching are reported in eTable 10. A total of 33 pwRRMS per treatment group contributed to the analysis. The median (IQR) follow-up time was 2.0 (1.7; 2.9) years for the ocrelizumab group and 2.0 (1.0; 2.9) years for the teriflunomide group. No significant differences were observed in the rates of DTI metrics changes across any of the regions of interest during the observation period (Table 3).

### PRL count

Baseline characteristics of the PRL cohort before and after propensity score matching are reported in eTable 11. A total of 31 pwRRMS per treatment group contributed to the analysis. The median (IQR) follow-up time was 1.5 (1.0; 2.2) years for the ocrelizumab group and 2.0 (1.2; 2.9) years for the teriflunomide group. During the observation period, three pwRRMS exhibited an increase in PRL count: two treated with teriflunomide and one with ocrelizumab. No patients showed resolution of PRLs. Longitudinal changes in PRL count did not differ between treatment groups (p = 0.96) (Table 3).

## Discussion

In this large observational study, we found no differences in the incidence of PIRA between pwRRMS treated with teriflunomide or ocrelizumab. Similarly, longitudinal changes in PRL burden and DTI metrics did not differ between the two groups. However, pwRRMS treated with teriflunomide exhibited reduced rates of whole brain and gray matter volume loss compared to those treated with ocrelizumab.

PIRA, a relatively recent clinical concept, has garnered considerable attention for highlighting that disability can accumulate independently of clinical relapses, even in pwRRMS with short disease duration and low disability [3, 4, 16]. This understanding has driven research into mechanisms underlying disease progression and underscored the urgent need for treatments targeting these processes [1, 2]. This is relevant as PIRA is associated with unfavorable long-term outcomes, particularly when it occurs early in the disease course [25].

Currently, data on the impact of DMTs on PIRA incidence remain limited. A pivotal analysis of the pooled OPERA I and II trials demonstrated that ocrelizumab reduced the risk of PIRA by 22% compared to interferon beta-1a [16]. Post-hoc analyses of the ASCLEPIOS I and II trials found teriflunomide significantly inferior to ofatumumab, which reduced the risk of PIRA by 56% [26]. However, comparative data on teriflunomide versus ocrelizumab have been lacking. Our study addresses this gap in an observational real-world cohort using propensity score-matched treatment groups and reveals no differences in PIRA incidence between the two treatments, suggesting comparable impacts on the mechanisms underlying disability progression. Despite potential biases in implementing the PIRA concept in observational studies, our study benefitted from regular clinical follow-up and used a specific definition for PIRA optimized for providing high specificity in the context of observational studies [3].

During follow-up, patients treated with teriflunomide exhibited significantly reduced BVL compared to those treated with ocrelizumab. In randomized controlled trials, teriflunomide has proved superior to placebo [27], not significantly different from ofatumumab and ublituximab [28, 29], and inferior to ponesimod [30] in reducing BVL. Ocrelizumab, on the other hand, demonstrated superiority over placebo in PPMS [31], though differences compared to interferon beta-1a were non-confirmatory in the OPERA I trial and non-significant in the OPERA II trial [32]. In our study, whole brain and gray matter atrophy rates were significantly higher in the ocrelizumab group. Conversely, regional analyses showed no differences in cortex or thalamus atrophy rates. While the results were robust after adjusting for additional confounders, statistical significance was lost in sensitivity analyses restricted to scans obtained after six months of treatment initiation. This loss of significance may suggest a potential influence of pseudoatrophy on the observed results. However, it is important to acknowledge that the sensitivity analyses were limited by reduced statistical power due to a smaller dataset, making direct comparisons challenging. Collectively, these findings suggest teriflunomide may exert a greater impact on reducing neurodegenerative processes leading to macroscopic BVL, though further data are needed to confirm this observation. Supporting our findings, a recent network meta-analysis of clinical trials found teriflunomide to be superior to placebo in reducing BVL, while this was not the case for ocrelizumab. Moreover, monoclonal antibodies overall did not appear to outperform most other DMTs in this domain [33].

No significant differences in DTI metrics were observed between treatment groups. Diffusion imaging examines the dynamics of water molecules, offering indirect insights into the microstructural organization of tissues [7, 8]. Among diffusion MRI approaches, DTI is the simplest model, providing measures sensitive to both macrostructural and microstructural tissue integrity, which reflect processes such as demyelination and axonal damage [8]. Despite its limitations—such as an inability to accurately model complex tissue architectures, particularly in regions with crossing fibers—DTI has been widely utilized in MS research. Quantitative maps derived from DTI have demonstrated clinical relevance, serving as biomarkers at both the lesional level and in normal-appearing tissue [34]. The lack of significant differences in longitudinal DTI metrics between treatment groups aligns with the observed lack of differences in clinical outcomes.

Similarly, there were no differences in PRL burden changes between groups. Data on DMT effects on PRLs remain limited, with existing studies suggesting that current DMTs have minimal impact on PRL resolution [35]. Observational studies have shown no reduction in PRL count with teriflunomide [36] or anti-CD20 therapies, including ocrelizumab [11]. Our findings are therefore consistent with these observations.

Our study has several limitations. First, its observational design and the non-random allocation of patients to treatments introduce potential bias. While we attempted to mitigate this by performing propensity score matching, incorporating various demographic, clinical, and paraclinical factors, the study design cannot eliminate residual bias, particularly given the differing characteristics of patients treated with teriflunomide and ocrelizumab in clinical practice. Moreover, some relevant clinical variables, such as the burden of spinal cord lesions, could not be included as matching criteria. Additionally, we did not include pre-enrollment ARR as a criterion for matching, opting instead for sNfL levels as a potentially more sensitive marker of inflammatory activity. Second, although the overall cohort size was large, the number of patients included in certain MRI analyses was relatively small, with a relatively short follow-up period; this limitation is due to the recent introduction of systematic 3D-EPI and diffusion MRI data collection in the SMSC study. Third, due to the limited sample size, we were unable to stratify patients further based on prior treatment sequences or systematically account for potential temporal lags in reaching treatment nadir. Fourth, given the exploratory nature of this study, we chose not to adjust for multiple comparisons, which may have increased the risk of type I errors. Fifth, PIRA was defined exclusively by changes in EDSS scores, without incorporating additional measures such as upper and lower limb dexterity, potentially overlooking treatment-specific impacts on these outcomes. Sixth, the follow-up period was unbalanced between groups in some analyses; however, to address this potential bias, we used mixed-effect models specifically designed to account for differences in follow-up duration.

Despite these limitations, our findings are of potential clinical relevance, demonstrating that teriflunomide is non-inferior to ocrelizumab in clinical and MRI measures reflecting smoldering disease activity in patients with RRMS. However, further data are needed to confirm these findings and better understand their long-term implications.

## Supplementary Information

Below is the link to the electronic supplementary material.

Supplementary file1 (DOCX 38 KB)

## Data Availability

The data that support the findings of this study are available on reasonable request.
