# Supplementary material for: Comparative effectiveness of teriflunomide and ocrelizumab on smoldering activity in multiple sclerosis: an observational study in the Swiss Multiple Sclerosis Cohort
Source: J Neurol. 2025 Jul 2;272(8):491. doi: 10.1007/s00415-025-13221-x (PMC12222313; doi:10.1007/s00415-025-13221-x)
Supplement: Supplementary file 1 — Supplementary file1 (DOCX 38 KB) [file 415_2025_13221_MOESM1_ESM.docx]

**Table of Contents**

- **eTable 1 – PRL cohort: MRI protocol**
- **eTable 2 – DTI cohort: MRI protocol**
- **eTable 3 – Atrophy cohort: MRI protocol**
- **eTable 4 - Clinical cohort: characteristics before and after matching**
- **eTable 5 - Atrophy cohort: characteristics before and after matching**
- **eTable 6 – Comparisons in longitudinal volumetric changes adjusting for baseline age, sex, and disease duration**
- **eTable 7 – Comparisons in longitudinal volumetric changes adjusting for annualized relapse rate**
- **eTable 8 – Comparisons in longitudinal volumetric changes in patients with ≥2 MRI time points**
- **eTable 9 – Comparisons in longitudinal volumetric changes including only scans performed ≥6 months after treatment start**
- **eTable 10 - DTI cohort: characteristics before and after matching**
- **eTable 11 - PRL cohort: characteristics before and after matching**

**eTable 1 – PRL cohort: MRI protocol**

|  | **Basel** | **Geneva** | **Lugano** |
| --- | --- | --- | --- |
| Nr. of brain MRI scans | 180 | 4 | 9 |
| Scanner vendor | Siemens | Siemens | Siemens |
| Scanner field strength | 3.0 T | 3.0 T | 3.0 T |
| Scanner model | Skyra (n=17);  Skyra_fit (n=163) | Prisma_fit (n=2);  Skyra (n=2) | Skyra (n=9) |

**eTable 2 – DTI cohort: MRI protocol**

|  | **Basel** | **Geneva** | **Lausanne** |
| --- | --- | --- | --- |
| Nr. of brain MRI scans | 180 | 21 | 16 |
| Scanner vendor | Siemens | Siemens | Siemens |
| Scanner field strength | 1.5 T (n=2); 3.0 T (n=178) | 3.0 T (n=21) | 1.5 T (n=1); 3.0 T (n=21) |
| Scanner model | Avanto (n=2); Skyra (n=18); Skyra_fit (n=160) | Prisma_fit (n=5);  Skyra (n=16) | MAGNETOM Sola (n=1); MAGNETOM Vida (n=1); Prisma_fit (n=6); Skyra (n=3); Skyra_fit (n=5) |

**eTable 3 – Atrophy cohort: MRI protocol**

|  | **Aarau** | **Basel** | **Bern** | **Geneva** | **Lausanne** | **Lugano** | **St. Gallen** |
| --- | --- | --- | --- | --- | --- | --- | --- |
| Nr. of brain MRI scans | 61 | 308 | 9 | 37 | 42 | 44 | 46 |
| Scanner vendor | Siemens | Siemens | Siemens | Siemens | Siemens | Siemens | Siemens |
| Scanner field strength | 1.5 T (n=48); 3.0 T (n=13) | 1.5 T (n=33); 3.0 T (n=275) | 1.5 T (n=6); 3.0 T (n=3) | 1.5 T (n=13); 3.0 T (n=24) | 1.5 T (n=1); 3.0 T (n=41) | 3.0 T (n=44) | 1.5 T (n=45); 3.0 T (n=1) |
| Scanner model | Avanto_fit (n=40); Espree (n=8); MAGNETOM Vida (n=4); Skyra (n=9) | Avanto (n=29); Avanto_fit (n=4); Prisma (n=1); Skyra (n=36); Skyra_fit (n=238) | Aera (n=2); Avanto_fit (n=4); MAGNETOM Vida (n=3) | Aera (n=11); MAGNETOM Sola (n=2); Prisma_fit (n=5); Skyra (n=19) | MAGNETOM Sola (n=1); MAGNETOM Vida (n=3); MAGNETOM Vida_fit (n=2); Prisma (n=3); Prisma_fit (n=15); Skyra (n=9); Skyra_fit (n=7) | Skyra (n=44) | Avanto_fit (n=45); Skyra (n=1) |

**eTable 4 - Clinical cohort: characteristics before and after matching**

|  | **Before matching** | | | **After matching** | | |
| --- | --- | --- | --- | --- | --- | --- |
|  | *Ocrelizumab* | *Teriflunomide* | *SMD* | *Ocrelizumab* | *Teriflunomide* | *SMD* |
| n | 495 | 128 |  | 128 | 128 |  |
| Age, mean (SD), years | 41.8 (12.0) | 48.2 (13.4) | 0.506 | 48.2 (12.0) | 48.2 (13.4) | 0.001 |
| Females, No. (%) | 325 (66) | 83 (65) | 0.017 | 82 (64) | 83 (65) | 0.016 |
| Disease duration, median [IQR], years | 8.8 [3.3;16.5] | 10.3 [4.2;19.0] | 0.187 | 11.7 [5.0;18.6] | 10.3 [4.2;19.0] | 0.028 |
| EDSS, median [IQR] | 2.5 [1.5;4.0] | 2.0 [1.5;3.5] | 0.153 | 2.5 [1.5;3.6] | 2.0 [1.5;3.5] | 0.070 |
| Number of previous DMTs, median [IQR] | 1 [0;3] | 2 [1;3] | 0.022 | 2 [0;3] | 2 [1;3] | 0.009 |
| Time under current treatment, median [IQR], years | 2.2 [0.0;5.1] | 2.3 [0.3;5.1] | 0.036 | 2.59 [0.0;6.1] | 2.3 [0.3;5.1] | 0.084 |
| sNfL, median [IQR], pg/ml | 10.6 [7.3;15.1] | 9.2 [7.1;11.9] | 0.134 | 9.9 [6.7;14.2] | 9.2 [7.1;11.9] | 0.026 |
| sNfL Z-score, mean (SD) | 0.88 (1.21) | 0.33 (1.15) | 0.463 | 0.33 (1.17) | 0.33 (1.15) | 0.004 |
| T2LV, median [IQR], ml | 8.2 [3.9;15.2] | 5.1 [2.4;9.3] | 0.293 | 6.8 [2.4;13.6] | 5.1 [2.4;9.3] | 0.084 |
| T2L count, median [IQR] | 34.0 [24.0;43.0] | 23.3 [17.0;33.5] | 0.482 | 27.0 [18.0;37.0] | 23.3 [17.0;33.5] | 0.036 |

*Abbreviations: DMTs = Disease-modifying therapies; EDSS = Expanded Disability Status Scale; IQR = interquartile range; SD = standard deviation; SMD = standardized mean difference; sNfL = serum neurofilament light chain; T2L = T2-lesion; T2LV = T2-lesion volume.*

**eTable 5 - Atrophy cohort: characteristics before and after matching**

|  | **Before matching** | | | **After matching** | | |
| --- | --- | --- | --- | --- | --- | --- |
|  | *Ocrelizumab* | *Teriflunomide* | *SMD* | *Ocrelizumab* | *Teriflunomide* | *SMD* |
| n | 105 | 72 |  | 72 | 72 |  |
| Age, mean (SD), years | 48.0 (12.1) | 49.8 (14.4) | 0.133 | 49.0 (12.3) | 49.8 (14.4) | 0.061 |
| Females, No. (%) | 71 (68) | 51 (71) | 0.070 | 48 (67) | 51 (71) | 0.090 |
| Disease duration, median [IQR], years | 12.6 [5.3;19.2] | 13.3 [5.0;22.4] | 0.134 | 13.8 [7.4;19.7] | 13.3 [5.0;22.4] | 0.034 |
| EDSS, median [IQR] | 2.5 [1.5;3.5] | 2.0 [1.5;3.5] | 0.096 | 2.5 [1.5;4.0] | 2.0 [1.5;3.5] | 0.128 |
| Number of previous DMTs, median [IQR] | 2 [0;3] | 2 [1;3] | 0.019 | 2 [0.75;3] | 2 [1;3] | 0.048 |
| Time under current treatment, median [IQR], years | 2.3 [0.0;5.8] | 2.3 [0.4;4.6] | 0.058 | 2.7 [0.3;5.9] | 2.3 [0.4;4.6] | 0.153 |
| sNfL, median [IQR], pg/ml | 9.7 [6.6;14.2] | 8.8 [6.9;13.2] | 0.160 | 10.0 [6.7;14.6] | 8.8 [6.9;13.2] | 0.103 |
| sNfL Z-score, mean (SD) | 0.33 (1.22) | 0.31 (1.21) | 0.013 | 0.38 (1.28) | 0.31 (1.21) | 0.051 |
| T2LV, median [IQR], ml | 6.1 [2.1;13.3] | 5.2 [2.4;11.3] | 0.066 | 6.4 [2.5;15.1] | 5.2 [2.4;11.3] | 0.023 |
| T2L count, median [IQR] | 25.7 [18.0;37.0] | 24.2 [16.6;34.6] | 0.039 | 24.2 [18.0;37.3] | 24.2 [16.6;34.6] | 0.006 |

*Abbreviations: DMTs = Disease-modifying therapies; EDSS = Expanded Disability Status Scale; IQR = interquartile range; SD = standard deviation; SMD = standardized mean difference; sNfL = serum neurofilament light chain; T2L = T2-lesion; T2LV = T2-lesion volume.*

**eTable 6 – Comparisons in longitudinal volumetric changes adjusting for baseline age, sex, and disease duration**

|  | **Ocrelizumab**  **(APC)** | **Teriflunomide**  **(APC)** | **Comparison** |
| --- | --- | --- | --- |
| n | 72 | 72 |  |
| BPF change | -1.16 [-1.40; -0.92] | -0.89 [-1.09; -0.69] | **p=0.024** |
| Cortical fraction change | -1.37 [-1.71; -1.03] | -1.09 [-1.36; -0.82] | p=0.09 |
| Thalamic fraction change | -1.04 [-1.36; -0.72] | -1.16 [-1.42; -0.91] | p=0.44 |
| GM fraction change | -1.32 [-1.61; -1.04] | -1.04 [-1.26; -0.81] | **p=0.040** |
| No. of MRI follow-ups, median [IQR] | 2.5 [2.0;3.0] | 2.0 [1.0;4.3] | SMD=0.295 |
| Follow-up time, mean (SD), years | 1.67 (1.26) | 2.15 (2.48) | SMD=0.246 |

Total number of scans included: 424.

Models included time, MRI protocol, TIV, as well as treatment group, baseline age, sex, and baseline disease duration and their interactions with time as covariates, and participants as random intercepts. Estimates were calculated for sex = female, age = 50 years, disease duration = 15 years.

Abbreviations: APC = annualized percentage change; BPF = brain parenchymal fraction; GM = gray matter; IQR = interquartile range; SD = standard deviation; SMD = standardized mean difference.

**eTable 7 – Comparisons in longitudinal volumetric changes adjusting for annualized relapse rate**

|  | **Ocrelizumab**  **(APC)** | **Teriflunomide**  **(APC)** | **Comparison** |
| --- | --- | --- | --- |
| n | 57 | 47 |  |
| BPF change | -1.11 [-1.33; -0.89] | -0.82 [-0.95; -0.69] | **p=0.020** |
| Cortical fraction change | -1.33 [-1.63; -1.02] | -1.01 [-1.19; -0.83] | p=0.07 |
| Thalamic fraction change | -1.08 [-1.39; -0.78] | -1.10 [-1.28; -0.93] | p=0.91 |
| GM fraction change | -1.27 [-1.54; -1.01] | -0.95 [-1.10; -0.79] | p=0.84 |
| No. of MRI follow-ups, median [IQR] | 3.0 [2.0; 4.0] | 4.0 [2.5; 6.0] | SMD=0.711 |
| Follow-up time, mean (SD), years | 2.11 (1.04) | 3.30 (2.37) | SMD=0.649 |

Total number of scans included: 384.

Models included time, MRI protocol, TIV, as well as treatment group and annualized relapse rate during the observation and their interactions with time as covariates, and participants as random intercepts. Estimates were calculated for annualized relapse rate = 0.15.

Abbreviations: APC = annualized percentage change; BPF = brain parenchymal fraction; GM = gray matter; IQR = interquartile range; SD = standard deviation; SMD = standardized mean difference.

**eTable 8 – Comparisons in longitudinal volumetric changes in patients with ≥2 MRI time points**

|  | **Ocrelizumab**  **(APC)** | **Teriflunomide**  **(APC)** | **Comparison** |
| --- | --- | --- | --- |
| n | 57 | 47 |  |
| BPF change | -1.05 [-1.24; -0.86] | -0.80 [-0.90; -0.69] | **p=0.025** |
| Cortical fraction change | -1.24 [-1.51; -0.97] | -0.96 [-1.12; -0.81] | p=0.09 |
| Thalamic fraction change | -0.97 [-1.23; -0.71] | -1.04 [-1.18; -0.89] | p=0.64 |
| GM fraction change | -1.20 [-1.43; -0.97] | -0.92 [-1.04; -0.79] | **p=0.034** |
| No. of MRI follow-ups, median [IQR] | 3.0 [2.0; 4.0] | 4.0 [2.5; 6.0] | SMD=0.711 |
| Follow-up time, mean (SD), years | 2.11 (1.04) | 3.30 (2.37) | SMD=0.649 |

Total number of scans included: 384.

Models included time, MRI protocol, TIV, treatment group, and the interaction between treatment group and time as covariates, and participants as random intercepts.

Abbreviations: APC = annualized percentage change; BPF = brain parenchymal fraction; GM = gray matter; IQR = interquartile range; SD = standard deviation; SMD = standardized mean difference.

**eTable 9 – Comparisons in longitudinal volumetric changes including only scans performed ≥6 months after treatment start**

|  | **Ocrelizumab**  **(APC)** | **Teriflunomide**  **(APC)** | **Comparison** |
| --- | --- | --- | --- |
| n | 56 | 46 |  |
| BPF change | -0.92 [-0.63; -1.22] | -0.72 [-0.59; -0.86] | p=0.23 |
| Cortical fraction change | -1.15 [-0.73; -1.56] | -0.82 [-0.63; -1.01] | p=0.17 |
| Thalamic fraction change | -0.96 [-0.56; -1.36] | -0.99 [-0.81; -1.17] | p=0.89 |
| GM fraction change | -1.17 [-0.81; -1.52] | -0.81 [-0.65; -0.97] | p=0.08 |
| No. of MRI follow-ups, median [IQR] | 3.0 [2.0; 4.0] | 4.0 [2.25; 6.0] | SMD=0.712 |
| Follow-up time, mean (SD), years | 2.14 (1.02) | 3.36 (2.36) | SMD=0.672 |

Total number of scans included: 265.

Models included time, MRI protocol, TIV, treatment group, and the interaction between treatment group and time as covariates, and participants as random intercepts.

Abbreviations: APC = annualized percentage change; BPF = brain parenchymal fraction; GM = gray matter; IQR = interquartile range; SD = standard deviation; SMD = standardized mean difference.

**eTable 10 - DTI cohort: characteristics before and after matching**

|  | **Before matching** | | | **After matching** | | |
| --- | --- | --- | --- | --- | --- | --- |
|  | *Ocrelizumab* | *Teriflunomide* | *SMD* | *Ocrelizumab* | *Teriflunomide* | *SMD* |
| n | 202 | 33 |  | 33 | 33 |  |
| Age, mean (SD), years | 42.1 (12.0) | 50.6 (13.5) | 0.664 | 47.7 (11.5) | 50.6 (13.5) | 0.235 |
| Females, No. (%) | 130 (64) | 27 (82) | 0.402 | 29 (88) | 27 (82) | 0.170 |
| Disease duration, median [IQR], years | 9.5 [3.9;17.6] | 9.5 [4.2;19.5] | 0.099 | 11.5 [4.8;20.3] | 9.5 [4.2;19.5] | 0.062 |
| EDSS, median [IQR] | 2.5 [1.5;4.0] | 2.0 [1.5;3.0] | 0.396 | 2.5 [1.5;3.2] | 2.0 [1.5;3.0] | 0.088 |
| Number of previous DMTs, median [IQR] | 1 [1;3] | 2 [0;2] | 0.144 | 2 [1;3] | 2 [0;2] | 0.080 |
| Time under current treatment, median [IQR], years | 2.3 [0.5;5.2] | 2.3 [0.0;5.4] | 0.019 | 2.6 [0.6;5.2] | 2.3 [0.0;5.4] | 0.022 |
| sNfL, median [IQR], pg/ml | 9.2 [6.7;14.3] | 9.9 [6.9;12.1] | 0.135 | 8.8 [5.8;12.8] | 9.9 [6.9;12.1] | 0.294 |
| sNfL Z-score, mean (SD) | 0.79 (1.30) | 0.30 (1.30) | 0.373 | 0.11 (1.42) | 0.30 (1.30) | 0.143 |
| T2LV, median [IQR], ml | 9.0 [4.1;16.1] | 5.1 [2.4;9.5] | 0.499 | 5.8 [1.7;10.1] | 5.1 [2.4;9.5] | 0.247 |
| T2L count, median [IQR] | 35.8 [24.0;48.9] | 20.1 [12.0;29.0] | 0.746 | 24.0 [18.9;32.5] | 20.1 [12.0;29.0] | 0.064 |

Abbreviations: DMTs = Disease-modifying therapies; EDSS = Expanded Disability Status Scale; IQR = interquartile range; SD = standard deviation; SMD = standardized mean difference; sNfL = serum neurofilament light chain; T2L = T2-lesion; T2LV = T2-lesion volume.

**eTable 11 - PRL cohort: characteristics before and after matching**

|  | **Before matching** | | | **After matching** | | |
| --- | --- | --- | --- | --- | --- | --- |
|  | *Ocrelizumab* | *Teriflunomide* | *SMD* | *Ocrelizumab* | *Teriflunomide* | *SMD* |
| n | 159 | 31 |  | 31 | 31 |  |
| Age, mean (SD), years | 42.6 (12.4) | 50.9 (13.1) | 0.651 | 49.8 (12.2) | 50.9 (13.1) | 0.085 |
| Females, No. (%) | 105 (66) | 23 (74) | 0.179 | 20 (65) | 23 (74) | 0.211 |
| Disease duration, median [IQR], years | 10.1 [3.6;17.6] | 10.0 [3.5;21.8] | 0.144 | 11.5 [4.7;21.5] | 10.0 [3.5;21.8] | 0.017 |
| EDSS, median [IQR] | 3.0 [2.0;4.0] | 2.5 [1.5;3.0] | 0.444 | 2.5 [1.75;3.75] | 2.5 [1.5;3.0] | 0.152 |
| Number of previous DMTs, median [IQR] | 2 [1;3] | 1 [0;2.5] | 0.137 | 2 [0.5;2] | 1 [0;2.5] | 0.042 |
| Time under current treatment, median [IQR], years | 2.3 [0.5;4.8] | 2.3 [0.0;5.3] | 0.047 | 2.2 [0.1;5.3] | 2.3 [0.0;5.3] | 0.082 |
| sNfL, median [IQR], pg/ml | 9.2 [6.7;13.8] | 8.8 [6.8;11.9] | 0.108 | 8.3 [6.5;13.3] | 8.8 [6.8;11.9] | 0.223 |
| sNfL Z-score, mean (SD) | 0.76 (1.24) | 0.20 (1.22) | 0.452 | 0.18 (1.20) | 0.20 (1.22) | 0.018 |
| T2LV, median [IQR], ml | 9.0 [4.2;16.9] | 4.9 [1.2;9.6] | 0.416 | 6.8 [2.3; 12.7] | 4.9 [1.2;9.6] | 0.077 |
| T2L count, median [IQR] | 37 [23.5;47.8] | 21 [14.4:35.0] | 0.578 | 26.8 [16.4;41.3] | 21 [14.4:35.0] | 0.101 |

Abbreviations: DMTs = Disease-modifying therapies; EDSS = Expanded Disability Status Scale; IQR = interquartile range; SD = standard deviation; SMD = standardized mean difference; sNfL = serum neurofilament light chain; T2L = T2-lesion; T2LV = T2-lesion volume
